# FGFRL1: Structure, Molecular Function, and Involvement in Human Disease

**DOI:** 10.3390/cimb47040286

**Published:** 2025-04-17

**Authors:** Lina Guan, Li Feng, Chaoli Wang, Yongen Xie

**Affiliations:** Institute of Basic Medicine Sciences and Forensic Medicine, North Sichuan Medical College, Fujiang Road, Nanchong 637000, China

**Keywords:** FGFRL1, structure, molecular function, human disease

## Abstract

FGFRL1 (fibroblast growth factor receptor-like 1) is a newly identified member of the FGFR family. Its extracellular domain resembles the four conventional FGFRs, while its intracellular part lacks the tyrosine kinase domain necessary for FGF-mediated signal transduction. At first, it was only considered a “decoy receptor”. However, recent studies have demonstrated that FGFRL1 is a multifunctional molecule involved in prenatal and postnatal growth of cartilage and osteogenesis, the development of embryonic kidney and diaphragm, the modulation of cellular biological behaviors, and cell signal transduction. The functional abnormalities of FGFRL1 contribute to human diseases including congenital disease, hypertension, osteoporosis, degenerative diseases of the central nervous system, and different kinds of tumors. The present review summarizes the research progress of FGFRL1, especially its subcellular location, molecular function, and associated human disease. These data may offer valuable resources for further studying the molecular function of FGFRL1 and disclosing the mechanism of its related human diseases.

## 1. Introduction

The fibroblast growth factors receptors (FGFRs) are members of the receptor tyrosine–protein kinase family. Four classic FGFR proteins have been identified, namely, FGFR1–FGFR4 [1,2]. The common structure of the classic FGFRs includes three extracellular immunoglobulin (Ig)-like domains, a single transmembrane helix, and an intracellular tyrosine kinase domain [3,4]. The extracellular portion of classic FGFRs possess binding sites with FGF ligands, heparin cofactors, and some related proteins [5,6]. After binding with corresponding FGF and heparin, the classic FGFRs dimerize and trans-autophosphorylate-specific tyrosine residues in the cytoplasmic domain of the receptors [7,8]. The signal is then transduced to the interior of the cell by various pathways, including Ras/MAP kinase, phospholipase Cγ, PI3-kinase, and STAT, which regulate a diverse variety of cellular functions such as cell proliferation, differentiation, migration, survival, and metabolism of target cells [9,10].

Fibroblast growth factor receptor-like 1 (FGFRL1) is the fifth member of the FGFR family [11,12]. It also contains three extracellular Ig-like domains and a single transmembrane sequence. However, it does not contain an intracellular protein tyrosine kinase domain but instead harbors a C-terminal domain of only 100 residues with undefined functions [13,14]. Through recognizing and binding to certain FGF ligands, FGFRL1 can positively or negatively modulate the cell signal transduction mediated by the classical FGFRs [15,16]. Gene mutation or aberrant expression of FGFRL1 is closely associated with a variety of human diseases including congenital disease hypertension, osteoporosis, degenerative diseases of the central nervous system, and different kinds of tumors [17,18,19,20].

## 2. Structural Features, Expression Patterns, and Subcellular Location

Human FGFRL1 was originally identified by Wiedemann et al. (2000) from a subtractive cDNA library established for screening cartilage-specific proteins [21]. Its molecular structure is quite similar to the four classical FGFRs. Sequence alignment and domain analysis indicate that FGFRL1 is a typical type I transmembrane protein with a single transmembrane helix. The extracellular part comprises three Ig-like domains termed Ig 1(D1), Ig2(D2), and Ig3(D3) and a linker region separating domains D1 and D2. The intracellular segment of FGFRL1 contains only 100 amino acids with a peculiar histidine-rich motif, which has no similarity to the intracellular tyrosine–protein kinase domain of all classical FGFRs [22,23].

The human FGFRL1 gene is located on chromosome 4p16.3 and consists of seven exons and five introns. The first exon contains only 50 base pairs of 5′-non-coding sequence (5′ UTR). The second to sixth exons sequentially encode the signal peptide, Ig1 domain, linker (acidic box), Ig2 domain, and Ig3 domain. The last exon encodes the transmembrane domain and the intracellular portion, and the 3′UTR is also contained within the last exon [21,24]. The human FGFRL1 gene is expressed in various tissues according to the tissue expression data in the GTEx database, with especially high levels of expression in the thyroid gland, adrenal gland, arteries, cerebral cortex, and salivary gland [15,25]. Currently, the FGFRL1 gene is found in all metazoans from amphioxus to mammals and birds, which have a single copy, whereas teleost fish have multiple *fgfrl1* genes that are believed to be the result of genome-wide duplication events [26,27,28]. FGFRL1 is expressed at very low levels in virtually all tissues of the vertebrate embryo and at relatively high levels in cartilage and muscle [29]. The amino acid sequence of FGFRL1 exhibits a relatively high conservation among different species. The chicken amino acid sequence shared 74% sequence identity (81% sequence similarity if conserved amino acid replacements were included) with the human and 72% identity with the rat sequence [14]. However, curiously enough, the amino acid sequence of the intracellular domain is barely conserved among different species, with the exception of three motifs, namely, a dileucine peptide, a tandem tyrosine-based motif (YxxLYxxI), and a histidine-rich sequence [30].

**Figure 1:**
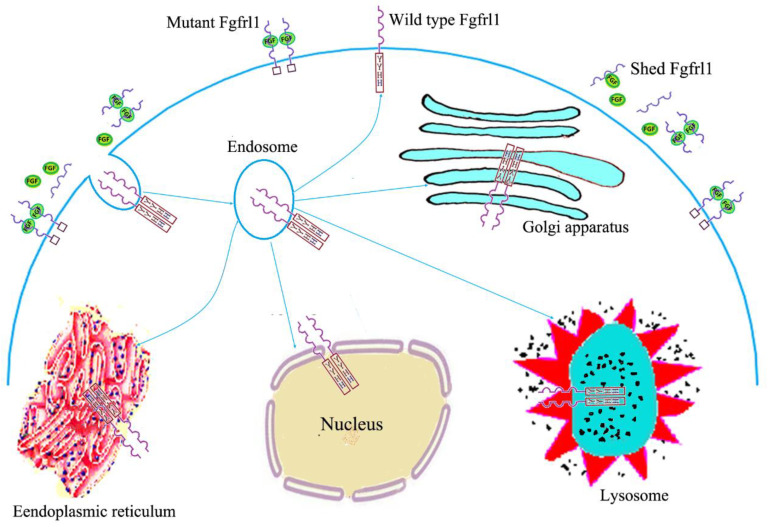
Subcellular location of FGFRL1. Cell membrane-bound wild-type FGFRL1 is internalized via endocytic events that result in the generation of an endosomal pool of FGFRL1. Endosomal FGFRL1 can be subsequently recycled to the plasma membrane or transported to the Golgi complex, endoplasmic reticulum, lysosome, and nuclear membrane. Mutant FGFRL1 is mainly located at the plasma membrane where it interacts with FGF ligands. FGFRL1 ectodomain can shed from the cell membranes and generate soluble receptors potentially capable of ligand scavenging.

Under physiological conditions, the majority of traditional FGFR (FGFR1~FGFR4) molecules are present on the cell surface. These FGFRs can be internalized via diverse endocytic events that result in the generation of an endosomal pool of receptors. These internalized receptor molecules can be subsequently degraded or recycled to the plasma membrane [31,32]. However, FGFRL1 exhibits an obviously different pattern of subcellular localization from the traditional FGFRs (Figure 1). A reporter gene assay showed that the wild-type FGFRL1 was preferentially located in the Golgi complex, the endoplasmic reticulum, and the nuclear membrane, while the mutant FGFRL1 was mainly found in the plasma membrane where it interacted with FGF ligands. Two motifs from the intracellular domain of FGFRL1 appeared to be responsible for this differential distribution, the tandem tyrosine-based motif and the histidine-rich sequence. Deletion of either one led to the preferential redistribution of FGFRL1 to the plasma membrane, suggesting that these two motifs function as signals for the trafficking of FGFRL1 from the plasma membrane to vesicular structures and the Golgi complex [33]. Thus, it seems like FGFRL1 reaches the plasma membrane but is efficiently and rapidly internalized via the vesicular transport systems and mutant FGFRL1 is retained at the cell surface for a longer period of time than the wild-type protein. In addition, it was proven that the FGFRL1 ectodomain can shed from the cell membranes of hepatocytes, HEK293 cells, and differentiating C2C12 myoblasts, generating soluble receptors potentially capable of ligand scavenging [34].

## 3. Molecular Function of FGFRL1

**Figure 2:**
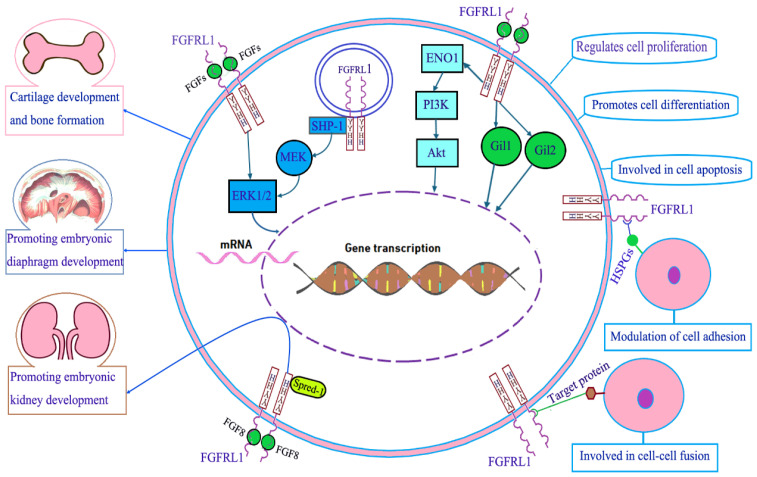
Molecular function of FGFRL1. FGFRL1 is involved in prenatal and postnatal growth of cartilage and osteogenesis and the development of embryonic kidney and diaphragm. FGFRL1 plays a role in promoting cell proliferation and cell differentiation. FGFRL1 can inhibit cell apoptosis. FGFRL1 promotes ENO1 expression and activates the PI3K/Akt signaling pathway. FGFRL1 activates the Hedgehog (Hh) signaling pathway and increases the downstream target genes (Gli1 and Gli2) expression. At insulin secretory granules, SHP-1 phosphatase can directly bind to the SH-2 motif in the short intracellular sequence of FGFRL1 and increase ERK1/2 protein phosphorylation in a ligand-independent manner. FGFRL1 at the plasma membrane can also bind extracellular FGF ligands to elevate ERK1/2 phosphorylation by means of an MEK-independent signaling cascade. FGF8/FGFRL1, spred-1, and some other molecules work in concert to control growth factor signaling during the development of the kidneys. The Ig2 domain can bind heparan sulfate proteoglycans (HSPGs) located on the cell surface of the neighboring cells and promote cell–cell adhesion. The Ig3 domain and transmembrane domain of FGFRL1 can bind the target protein of the adjacent cells and mediate cell fusion.

With an ectodomain closely resembling the traditional FGFRs, FGFRL1 was proven to bind some FGFs both in its soluble and in its membrane-bound state. Ectopic expression of FGFRL1 in Xenopus embryos antagonized FGFR signaling during early development [34]. Therefore, FGFRL1 was initially regarded as a decoy receptor that inhibited canonical FGFR ligand-induced signaling [16]. In the past few decades, the unique structure of FGFRL1 has aroused great interest in the study of its molecular function. The collective evidence suggests that FGFRL1 is involved in prenatal and postnatal growth of cartilage and osteogenesis, the development of embryonic kidney and diaphragm, the modulation of cellular biological behaviors, and cell signal transduction (Figure 2).

### 3.1. FGFRL1 in Cartilage Development and Bone Formation

FGFRL1 as a newly identified FGFR is initially cloned from cartilage tissues. The role of FGF in cartilage development and bone formation has naturally attracted the attention of researchers. Zebrafish has two copies of *fgfrl1* genes, *fgfrl1a* and *fgfrl1b*. It is proven that both duplicates, *fgfrl1a* and *1b*, are required for the proper formation of ceratobranchial cartilage, while *fgfrl1b* appears to exert a broader role in the development of all pharyngeal and cranial cartilage. Subsequent studies reveal that Fgfrl1-depleted zebrafish embryos do not express the transcription factor glial cells missing 2 (gcm2), a gene necessary for cartilage and gill filament formation, in the ectodermal lining of the branchial arches. In addition, two transcription factors essential for chondrogenesis, sox9a and runx2b, fail to express within the mesenchymal condensation of the branchial arches [35,36]. In a 16-day-old mouse embryo, the FGFRL1 mRNA was detected in cartilaginous structures, such as the primordia of bones and the permanent cartilage of the trachea, the ribs, and the nose. The expression of FGFRL1 was also detected in the resting area of the postnatal proximal tibial growth plate in rats [37]. Transcriptional profiling was used to evaluate changes in gene expression of articular chondrocytes between neonatal and adult horses. It is revealed that FGFRL1 mRNA is significantly upregulated in adult horses compared to the foals, suggesting that FGFRL1 may be involved in cartilage homeostasis in adults [38]. Therefore, it can be inferred that FGFRL1 not only participates in embryonic cartilage formation but also plays an important regulatory role in postnatal osteogenesis. In humans, FGFRL1 mutation or deletion was proven to be associated with skeletal malformations and osteoporosis [39]. All in all, FGERL1 has proven to be essential for cartilage development and bone formation.

### 3.2. FGFRL1 in Diaphragm Development

To explore the function of the Fgfrl1 receptor, Baertschi et al. (2007) [40] created mice with a targeted disruption of the *fgfrl1* gene. These mice developed normally until term but died within a few minutes after birth due to respiratory failure. Although examination under a stereomicroscope revealed normally developed lungs of regular size, the lungs from *fgfrl1−/−* mice were barely inflated. Subsequent studies show that the respiratory problem is caused by a significant reduction in the size of the diaphragm muscle, which is not sufficient to inflate the lung after birth. In *fgfrl1−/−* animals, the diaphragm sealed the abdominal cavity tightly from the thoracic cavity as in wild-type animals. However, the diaphragms of *fgfrl1−/−* mice were significantly thinner than those of the wild-type animals. On closer inspection of dissected diaphragms, it was found that a general hypotrophy of the diaphragm muscle is the underlying cause for the respiratory problems of the *fgfrl1* knockout mice. When other skeletal muscles were inspected, no significant differences could be detected between control and *fgfrl1−/−* mice. In a nitrofen model of congenital diaphragmatic hernia (CDH), decreased expression of FGFRL1 was detected during the later gestational stages of the mice [41]. Gofin et al. (2021) [42] also found that two FGFRL1 missense variants contributed to congenital diaphragmatic hernia development in humans. From these data, it can be inferred that FGFRL1 plays a critical role in the development of the diaphragm. In a subsequent study, Amann et al. (2014) [43] demonstrated that the diaphragm of *fgfrl1* knockout animals lacked any slow muscle fibers at embryonic day 18.5 (E18.5), as indicated by the absence of slow fiber markers Myh7, Myl2, and Myl3. In contrast to slow fibers, fast fibers do not appear to be affected, as shown by the expression of fast fiber markers Myh3, Myh8, Myl1, and MylPF. Why is the absence of muscle fibers caused by the FGFRL1 mutation only observed in slow fibers but not in fast fibers? The fundamental mechanisms need to be elucidated in future studies.

### 3.3. FGFRL1 in Kidney Development

When systematically screening the *fgfrl1−/−* embryos for organ defects, Gerber et al. (2009) [44] discovered that the metanephric kidneys were missing in all of the examined animals. On embryonic day 18.5 (E18.5), only a small, rudimentary structure that was attached to the ureter was found in place of the kidney. The rest of the urogenital system, including the testes, ovaries, bladder, and ureter, appeared to be normally developed. A dramatic reduction in ureteric branching morphogenesis and a lack of mesenchymal-to-epithelial transition were observed in the nephrogenic mesenchyme during the development of the *fgfrl1−/−* embryos. FGFRL1 seems to be an essential factor for mesenchymal differentiation and the formation of epithelial renal vesicles in the early steps of nephrogenesis. In a recent study, mice with deletions of the individual domains of FGFRL1 were generated. Mice lacking the intracellular domain were viable and phenotypically normal. Mice lacking the Ig1 domain were also viable and normal but had a reduced life span. Mice lacking the Ig2 or the Ig3 domain were born alive but died within 24 h after birth. Ig2-deficient animals exhibited substantially smaller kidneys than wild-type littermates and contained a lower number of glomeruli. Ig3-deficient mice completely lacked metanephric kidneys. These results suggest that regulating nephrogenesis is primarily accomplished by the Ig3 domain with some contribution from the Ig2 domain [45]. Further investigations reveal that FGFRL1 knockout mice lack metanephric kidneys and do not express Lhx1, Wnt4, and Fgf8, three important regulators of the mesenchymal-to-epithelial transition. Moreover, the phenotype of conditional Fgf8 knockout mice is quite similar to that of FGFRL1 knockout mice [46,47]. Therefore, it can be inferred that the binding of FGF8 to FGFRL1 may play an important role in driving the formation of nephrons in the developing kidney.

### 3.4. FGFRL1 in Cell Proliferation, Cell Differentiation, and Cell Apoptosis

The initial study indicates that FGFRL1 has antiproliferative effects. When the coding sequence of FGFRL1 was cloned into a eukaryotic expression vector and transfected into MG-63 cells, the transfected cells revealed a significant reduction in cell proliferation. No reduction was observed with cells that had been transfected with the empty vector [14]. Guan et al. also found that FGFRL1 overexpression inhibited the proliferation of colon cancer HCT116 cells. However, more and more studies have proven that FGFRL1 can promote cell proliferation [48]. Tsuchiya et al. demonstrated that FGFRL1 increased the proliferation of esophageal squamous cell carcinoma (ESCC) cells by inhibiting cell cycle arrest in the G1/G0 phase [49]. Zuo et al. (2015) identified that the downregulation of FGFRL1 with miRNA 210 inhibited the proliferation of SCC10A cells by inducing cell cycle arrest [50]. It was also found that the knockdown of FGFRL1 significantly inhibited cell proliferation in ovarian cancer (OC) cells [51]. Thus, it seems like FGFRL1 promotes cell proliferation in most cases.

FGFRL1 has an obviously positive effect on cell differentiation. It is revealed that FGFRL1 is involved in the differentiation of C2C12 cells in vitro [40]. When C2C12 myoblasts differentiate into myotubes, FGFRL1 is barely expressed during the proliferative stage, but its expression is significantly upregulated once proliferation has ceased and cell differentiation has initiated. The role of FGFRs was analyzed in the process of mesenchymal stromal cell (MSC) differentiation with gene microarray and qRT-PCR. FGFRL1 mRNA expression strongly increased during MSC differentiation to osteoblasts. FGFR1 knockdown inhibited osteoblast differentiation, which was accompanied by a decrease in FGFRL1 expression in osteoblasts [52]. FGFRL1 is also involved in the differentiation of mesenchymal nephron precursor cells into tubular epithelial structures. FGFRL1 mRNA is expressed in the metanephric mesenchyme, with the strongest expression observed in differentiating renal vesicles and nascent nephrogenic structures [44]. Therefore, FGFRL1 expression is always consistent with the differentiation state of the cells.

Most of the FGFs and classic FGFRs are demonstrated to exhibit anti-apoptosis effects [53,54]. Several studies have proven that FGFRL1 is also involved in cell apoptosis. Targeted disruption of the *fgfrl1* gene in mice leads to severe renal dysgenesis and an increase in apoptosis in the cortical zone of the remnant kidney [44]. It was observed that the silencing of FGFRL1 increased the apoptosis rate of ovarian cancer (OC) cells [51]. Consistent with this, caspase-3/7 activity in OC cells was significantly increased by the silencing of FGFRL1. The downregulation of FGFRL1 was also proven to induce cell cycle arrest and apoptosis in human esophageal squamous cell carcinoma (ESCC) cell lines and human small cell lung cancer (SCLC) cell lines [49,55]. Thus, the current data suggest that FGFRL1 may act as an inhibitor in cell apoptosis.

### 3.5. FGFRL1 in Cell Adhesion and Cell Fusion

FGFRL1 has been demonstrated to be involved in the modulation of cell–cell adhesion. FGFRL1 is found to be enriched at cell–cell contact sites. When coated on the bacterial plastic dishes, the extracellular portion of the FGFRL1 protein (Ig1-Ig3 domains) promoted the adhesion of various cell lines including MG63, A204, and MC3T3-E1 [56]. The adhesion-promoting effect was further verified in HEK293-TetOn cells that express FGFRL1ΔC with the pTRE expression vector, which harbors a tetracycline-responsive promoter element. No obvious cell–cell adhesion was able to be observed in the absence of doxycycline. However, when supplemented with doxycycline, the cells began to form cell–cell adhesion and cell clusters. It is revealed that this adhesion-promoting activity is mediated by heparan sulfate proteoglycans (HSPGs) located on the cell surface of cultivated cells since the adhesion can be blocked with soluble heparin [57].

Some researchers showed that FGFRL1 induced fusion of cultured CHO cells into large syncytia comprising several hundred nuclei [58]. Peculiar net-like structures with pores of about 1 μm in diameter are preferentially found in the membrane area where two cells contact each other. It is possible that these membrane regions with fusion pores represent structures that set in motion the cell–cell fusion process [59]. A reporter gene assay demonstrated that the Ig3 domain and the transmembrane domain of FGFRL1 are both necessary for its fusion-promoting activity. A hydrophobic pocket in the extracellular Ig3 domain of FGFRL1 appears to interact with the target protein of neighboring cells, and four amino acids (L281, F303, L339, and V304) located in the hydrophobic site of the Ig3 domain are required for the target protein interaction [60].

### 3.6. FGFRL1 in Cell Signal Transduction

It is demonstrated that FGFRL1 can bind to FGF2, FGF3, FGF4, FGF8, FGF10, FGF18, and FGF22. Through interacting with these FGFs, FGFRL1 can modulate the function of the FGF/FGFR signaling pathway [61]. For example, FGFRL1 is proven to interact with FGF8 and plays an important role in early kidney development. Although FGF8 was verified to bind to the Ig2 domain of FGFRL1, the downstream signaling mechanism of FGF8/FGFRL1 binding is not clear. One speculation is that FGF8/FGFRL1 may interact with a third molecule and trigger corresponding signaling transduction. Another explanation is that FGFRL1 might restrict the signaling range of FGF8 to the region of the renal vesicle [62]. Recent studies have demonstrated that FGFRL1 is involved in several cell signaling pathways in FGF ligands in a dependent or independent manner. FGFRL1 can promote ENO1 expression and activate the PI3K/Akt signaling pathway that regulates cell division, differentiation, and apoptosis, and it is one of the most frequently altered signaling pathways in cancer [63,64]. FGFRL1 can activate the Hedgehog (Hh) signaling pathway and increase the downstream target genes (Gli1 and Gli2) expression. Hh signaling plays an essential role in embryonic development and also in tissue and organ homeostasis in adults [51,65]. At insulin secretory granules, SHP-1 phosphatase can directly bind to the SH-2 motif in the short intracellular sequence of FGFRL1 and increase ERK1/2 protein phosphorylation in a ligand-independent manner. FGFRL1 at the plasma membrane can also bind extracellular FGF ligands to elevate ERK1/2 phosphorylation by means of an MEK-independent signaling cascade. The ERK1/2 signaling modulates cell proliferation, migration, differentiation, and survival [66]. In addition, a Sprouty/Spred family protein, Spred-1, was demonstrated to bind the C-terminal histidine-rich sequence of FGFRL1 and increase the retention time of FGFRL1 at the plasma membrane. It is speculated that FGFRL1, Spred-1, and some other proteins work in concert to control growth factor signaling during the development of the kidneys and other organs [67,68].

## 4. FGFRL1 and Human Disease

### 4.1. Congenital Disease

Antley–Bixler syndrome (ABS) is the first congenital disease that was found to be associated with FGFRL1 mutation. A British patient was identified as harboring a frameshift mutation in exon 6 shortly before the end of the open reading frame of the FGFRL1 gene. The mutation causes an elongation of the encoded protein by 47 residues before a stop codon is reached at amino acid position 551. This patient was diagnosed with ABS and presented with craniosynostosis, radio-ulnar synostosis, and genital anomalies. Further study reveals that the mutant FGFRL1 contributes to the skeletal malformations of the patient [33].

Wolf–Hirschhorn syndrome (WHS) is another congenital disease associated with FGFRL1 deletion. WHS is caused by deletions in the short arm of chromosome 4 (4p) and occurs in about 1 per 20,000 births [69,70]. Catela et al. (2009) demonstrated that targeted deletion of the mouse *fgfrl1* gene recapitulated a broad array of WHS phenotypes, including abnormal craniofacial development, axial and appendicular skeletal anomalies, and congenital heart defects [71]. Deletion of the *fgfrl1* gene was also detected in all the WHS patients with facial dysmorphic features [72,73]. Therefore, *fgfrl1* gene deletion may contribute to part of the facial characteristics of WHS in 4p16.3 deletion patients.

In addition, congenital diaphragmatic hernia (CDH), familial gastroschisis, and congenital heart disease are also demonstrated to be associated with FGFRL1 mutation or variation [74,75,76].

### 4.2. Hypertension and Osteoporosis

FGF can modulate blood pressure and bone mineral density, which was first found in the study of giraffe FGFRL1. The giraffe FGFRL1 gene was found to have seven unique amino acid substitutions, which were not found in any other ruminant. Moreover, gene-edited mice with the giraffe-type FGFRL1 showed exceptional hypertension resistance and higher bone mineral density. These results suggest that giraffe-type FGFRL1 may modulate blood pressure and bone mineral density [77]. Subsequently, several research groups investigated the association of FGFRL1 with hypertension and osteoporosis in humans and demonstrated that single-nucleotide polymorphism (SNP) of the *fgfrl1* gene is closely related to hypertension and osteoporosis. Four SNPs (rs16998073, rs13143527, rs55639339, and rs10010999) were significantly associated with hypertension. Six SNPs (rs13143527, rs55639339, rs74921869, rs35220088, rs73070422, and rs78590462) were significantly associated with osteoporosis. Two SNPs, rs13143527 and rs55639339, were associated with both hypertension and osteoporosis. Rs55639339 showed an increased risk for hypertension and osteoporosis, whereas the rs13143527 variant was associated with a decreased risk of hypertension and osteoporosis [78,79,80].

### 4.3. Degenerative Diseases of the Central Nervous System

It is well known that the SNCA_A53T_ mutation causes inherited familial Parkinson’s disease (PD). In order to explore the mechanisms underlying selective dopaminergic neurodegeneration in PD, cell and animal models carrying the SNCA_A53T_ mutation were constructed to analyze the differentiated expression genes. The upregulation of FGFRL1 was found both in the SNCA_A53T_ knockin dopaminergic neuron cell lines and in the dopaminergic neurons of the SNCA_A53T_ transgenic mice. Furthermore, the knockdown of FGFRL1 rescued oxidative stress-induced cell death in dopaminergic cells bearing SNCAA53T mutation. Therefore, FGFRL1 may contribute to dopaminergic neurodegeneration in PD [81]. However, the exact mechanism by which FGFRL1 promotes the degeneration of dopaminergic neurons is still unclear and needs further study. Comorbidity exists between amyotrophic lateral sclerosis (ALS) and PD; for example, α-synuclein is primarily associated with PD, evidence that also supports its involvement in ALS pathogenesis by accelerating the oligomerization of several ALS-causing proteins [82]. Using bioinformatic analysis, *fgfrl1* was demonstrated as one of the comorbid genes between ALS and PD [83].

### 4.4. FGFRL1 and Cancer

It has been reported that abnormal FGFRL1 protein expression is closely associated with the development, progression, and invasion of various tumors. In most cases, FGFRL1 exhibits to promote the development and progression of tumors including bladder cancer, esophageal cancer, larynx carcinoma, lung cancer, ovarian cancer, and prostate cancer. However, in some types of cancer, such as osteosarcoma and pancreatic cancer, FGFRL1 appears to inhibit the progression and invasion of cancer (Table 1).

#### 4.4.1. Bladder Cancer

Loss of heterozygosity (LOH) of chromosome arm 4p is a common event in bladder and other malignancies [84]. FGFRL1, which maps within this region, is speculated as a deletion target. However, di Martino et al. (2013) [85] demonstrated that the average FGFRL1 protein expression in bladder cancer tissue is independent of 4p16.3 LOH status. Yang et al. (2017) [86] observed that FGFRL1 was overexpressed in bladder cancer tissues and bladder cancer cell lines as compared with para-tumor normal tissues and human uroepithelial cells SV-HUC-1. They also discovered that miR-210-3p was significantly downregulated in bladder cancer tissue compared to the para-tumor normal tissue. A dual-luciferase reporter assay showed that miR-210-3p could directly inhibit the expression of FGFRL1 by binding to the 3′-UTR of its mRNA. With miR-210-3p overexpression in bladder cancer cell lines, cell proliferation, invasion, and migration decreased significantly, and tumor growth in vivo was suppressed. All these results indicate that FGFRL1 can promote bladder cancer growth and metastasis in vitro and in vivo, and miR-210-3p plays an important role in the inhibition of bladder cancer growth and metastasis through targeting FGFRL1.

#### 4.4.2. Esophageal Cancer

Sixty-nine patients with esophageal squamous cell carcinoma (ESCC) were evaluated for FGFRL1 expression by tissue microarray and compared with the clinicopathological factors of patients. It was found that FGFRL1 was positively associated with lymph node metastasis and tumor growth in the patients. The results also showed that the prognosis of the FGFRL1-positive patients was significantly worse than that of the FGFRL1-negative patients. However, FGFRL1 expression was not an independent prognostic factor for the patients [87]. Further analysis revealed that patients who tested negative for the expression of both FGFRL1 and FGFR4 had the best prognosis, while patients who co-expressed FGFRL1 and FGFR1 had the worst prognosis [88]. Tsuchiya S et al. (2011) [49] identified FGFRL1 as a target of miR-210 in ESCC and demonstrated that FGFRL1 accelerates cancer cell proliferation by preventing cell cycle arrest in G(1)/G(0). The immunofluorescence assay revealed increased expression of FGFRL1 from well-differentiated ESCC cells to poorly differentiated ESCC cells [89]. To further examine the effects of FGFRL1 in vivo, mice were injected subcutaneously with wild-type and FGFRL1-deficient KYSE520 cells. Tumors derived from FGFRL1-deficient cells exhibited reduced growth in vivo compared to those from parental KYSE520 cells. The hematoxylin and eosin staining showed that FGFRL1-deficient cells formed well-differentiated squamous cell carcinomas in vivo, whereas wild-type cells formed moderately differentiated squamous cell carcinomas. Microarray analysis of mRNA expression revealed that FGFRL1 depletion resulted in a decreased expression of proteins associated with motility and an invasion of tumor cells [90]. These results indicate that FGFRL1 promotes the progression and metastasis of esophageal cancer, and FGFRL1 is closely associated with the prognosis of esophageal cancer patients.

#### 4.4.3. Larynx Carcinoma

Tumor microenvironment hypoxia has been shown to be a negative prognostic factor for most solid tumors, associated with increased metastasis and decreased overall survival [91]. Hypoxia regulates the expression of some genes that are sensitive to oxygen pressure. Hypoxia-inducible factor-1 (HIF-1), which is induced by low oxygen pressure, can subsequently influence the expression of a number of genes and microRNAs. miR-210 is a main downstream effector gene of HIF-1, which is upregulated in several types of solid tumors [92,93] It was found that the FGFRL1 protein level decreased when miR-210 was overexpressed in larynx carcinoma cell line SCC10A. In addition, the expression of miR-210 inhibited the proliferation of SCC10A cells and repressed tumor xenograft growth in vivo. Moreover, the overexpression of FGFRL1 effectively released the miR-210-induced suppression of SCC10A cell proliferation [50]. These results indicate that FGFRL1 may promote the progression of larynx carcinoma.

#### 4.4.4. Lung Cancer

Fan et al. (2020) [94] found that long non-coding RNA FGD5-AS1 promotes non-small cell lung cancer (NSCLC) cell proliferation through sponging hsa-miR-107 to upregulate FGFRL1 expression. The results of clinical sample detection show that FGFRL1 is overexpressed in small-cell lung cancer (SCLC) tissues, and high FGFRL1 expression is associated with the clinical stage, chemotherapy response, and survival time of SCLC patients. Further studies demonstrated that FGFRL1 levels are significantly upregulated in multidrug-resistant SCLC cells compared with the sensitive parental cells. Knockdown of FGFRL1 in chemoresistant SCLC cells increased chemosensitivity by increasing cell apoptosis and cell cycle arrest, whereas the overexpression of FGFRL1 in chemosensitive SCLC cells produced the opposite results [64]. An RNA-binding protein HuR has been demonstrated to mediate the chemoresistance of SCLC by regulating FGFRL1 expression [55]. In addition, Wang et al. (2020) [95] found that lung cancer cells overexpressing FGFRL1 showed decreased metastatic ability, whereas silencing FGFRL1 increased the metastatic ability of lung cancer cells. These results indicate that FGFRL1 is related to the progression, chemoresistance, and prognosis of lung cancer.

#### 4.4.5. Osteosarcoma

Several studies have demonstrated that miR-210 can negatively regulate FGFRL1 expression by directly targeting the 3′-untranslated region of FGFRL1 mRNA [49]. Liu et al. found that the expression of miR-210 is highly elevated while FGFRL1 expression is reduced inversely in osteosarcoma tissues compared with matched normal tissues. The results of trans-well assays show that miR-210 promotes osteosarcoma cell migration and invasion, while FGFRL1 overexpression deprives the promotion effect of miR-210 on cell migration and invasion [96]. When overexpressed in MG-63 osteosarcoma cells, FGFRL1 showed a negative effect on cell proliferation [14]. These results suggest that FGFRL1 may inhibit the development and progression of osteosarcoma.

#### 4.4.6. Ovarian Cancer

Through screening of 241 different human tumors with the help of a profiling array and quantitative PCR, it is revealed that FGFRL1 aberrant expression might contribute to the development and progression of ovarian cancer (OC) [97]. To discover ovarian tumor-specific molecules, Barrett et al. developed custom bioinformatics algorithms to analyze transcriptome sequence data of 296 ovarian cancer and 1,839 normal tissues and validated putative tumor-specific mRNA isoforms by RT–qPCR. It was found that 15 isoforms, including FGFRL1, which was expressed in high-grade serous ovarian (HGS-OvCa) tumors, were not expressed in the ovary or fallopian tube [98]. A recent study has proven that FGFRL1 is significantly upregulated in both OC cells and tissues compared with the normal controls, and high FGFRL1 expression is correlated with poor prognosis in OC patients. The downregulation of FGFRL1 significantly inhibits cell proliferation and migration of OC cells in vitro. Loss of function of FGFRL1 increases the apoptosis rate of OC cells accompanied by increased caspase-3/7 activity. The weight and size of tumors formed by FGFRL1-siRNA transfected cells are significantly decreased in comparison with the tumors formed by the siRNA–control transfected cells in a nude mice transplant tumor model [51]. Therefore, FGFRL1 is demonstrated as a crucial factor in the development, progression, and prognosis of human OC, indicating that it is a novel therapeutic target that can be used for the treatment of OC.

#### 4.4.7. Pancreatic Cancer

Guo et al. (2024) [99] observed that miR210 is expressed in pancreatic cancer (PC) stem cell-derived exosomes. Through targeting FGFRL1, miR210 mimics promoted M2 polarization of macrophages, while FGFRL1 overexpression inhibited miR210-mediated M2 polarization. When M2-type macrophages cocultured with pancreatic cancer cells and were treated with gemcitabine, the migration rate and gemcitabine resistance of PC cells increased significantly compared to PC cells cocultured with M0-type macrophages and treated with gemcitabine. In a PC cell xenograft animal model, the M2-type macrophage promoted xenograft tumor progression, which was associated with the PI3K/AKT/mTOR pathway activation and drug resistance protein upregulation. Therefore, it seems like FGFRL1 downregulation promotes the progression and chemoresistance of pancreatic cancer by promoting M2 polarization of macrophages.

#### 4.4.8. Prostate Cancer

Yu et al. (2022) [100] demonstrated that FGFRL1 was significantly upregulated in prostate cancer (PCa) tissues compared to adjacent nonmalignant prostate tissues. Statistical analysis of clinical data and immunohistochemical (IHC) staining showed the relocalization of membranous FGFRL1 in nonmalignant prostate to cytoplasmic and nuclear sites in PCa. The level of membranous FGFRL1 was negatively associated with high Gleason scores (GSs) and Ki67, while increased cytoplasmic and nuclear FGFRL1 showed a positive correlation. Cox regression analysis indicated that nuclear FGFRL1 was an independent prognostic marker for biochemical recurrence after radical prostatectomy. In accordance with clinical data, FGFRL1 knockout markedly suppressed the *growth* of *PC3M cell xenograft* tumors. Wu et al. (2023) [101] found that FGFRL1 was significantly upregulated in PCa cells compared to the prostate epithelial cell line RWPE-1. Long non-coding RNA VPS9D1-AS1 was found to upregulate FGFRL1 by competitively sponging miR-187-3p to accelerate the malignant behaviors of PCa cells. These results indicate that FGFRL1 upregulation and altered cellular compartmentalization contribute to PCa development and progression, and the nuclear FGFRL1 could serve as a prognostic marker for PCa patients.

**Table 1:** The expression level, the influence on cancer cell’s biological behaviors of FGFRL1, and its roles in cancer progression, chemoresistance, and prognosis.

Cancers	Expression Level	Influence on Cancer Cell’s Biological Behaviors	Roles in Cancer Progression, Chemoresistance, Metastasis, and Prognosis	(Refs.)
Bladder cancer	Upregulated in bladder cancer cell lines and tissue samples	FGFRL1 downregulation inhibits the proliferation, migration, and invasion of bladder cancer cells	Promotes bladder cancer growth and metastasis	[85,86]
Esophageal cancer	Either positive or negative expression can be detected in esophageal cancer tissue	FGFRL1 accelerates cancer cell proliferation and invasion	FGFRL1 promotes the progression and metastasis of esophageal cancer. FGFRL1-positive is correlated with poor prognosis in esophageal cancer patients	[87,88]
Larynx carcinoma	None	Overexpression of FGFRL1 promotes the proliferation of larynx carcinoma SCC10A cells	Promotes the progression of larynx carcinoma	[50]
Lung cancer	Upregulated in small-cell lung cancer (SCLC) tissues and multidrug-resistant SCLC cells	FGFRL1 accelerates cancer cell proliferation. Knockdown of FGFRL1 increases the chemosensitivity of chemo-resistant SCLC cells	Promotes the progression and chemoresistance of lung cancer	[64,94]
Osteosarcoma	Downregulated in osteosarcoma tissues compared with matched normal tissues	Overexpression of FGFRL1 inhibits osteosarcoma cell migration and invasion	Inhibits the progression of osteosarcoma	[14,96]
Ovarian cancer (*OC*)	Upregulated in both OC cells and tissues compared to the normal controls	Downregulation of FGFRL1 inhibits the proliferation and migration of OC cells	Promotes the development and progression of OC. FGFRL1 upregulation is correlated with poor prognosis	[51,97]
Pancreatic cancer (PC)	None	Inhibits the migration and chemoresistance of PC cells through inhibiting M2 polarization of macrophages	Inhibits the progression and chemoresistance of pancreatic cancer	[99]
Prostate cancer (PCa)	Upregulated in PCa tissues and PCa cells compared to the corresponding normal controls	Upregulation of FGFRL1 accelerates the malignant behaviors of PCa cells	Promotes the development and progression of PCa	[100,101]

In addition, FGFRL1 was also shown to be associated with hepatocellular carcinoma [102], oral squamous cell carcinoma [103], and rectal cancer [104], but the associated research data are relatively limited.

## 5. Conclusions

As a newly identified receptor of the FGFR family, Fgfrl1 has a distinctive molecular structure at the intracellular part as compared with the traditional FGFRs. Fgfrl1 is not only a “decoy receptor” but also an active receptor with multiple functions. Current studies have proven that Fgfrl1 is involved in prenatal and postnatal growth of cartilage and osteogenesis, the development of embryonic kidney and diaphragm, the modulation of cellular biological behaviors, and cell signal transduction. However, many aspects of the exact molecular mechanism by which Fgflr1 exerts its biological function still remain unclear. Gene mutation and abnormal expression of Fgfrl1 are demonstrated to correlate with human disease including congenital disease, hypertension, osteoporosis, degenerative diseases of the central nervous system, and different kinds of tumors. FGFRL1 is closely associated with the development, progression, invasion, drug resistance, and prognosis of several types of cancer, such as bladder cancer, esophageal cancer, larynx carcinoma, lung cancer, ovarian cancer, pancreatic cancer, and prostate cancer. Its aberrant expression is a potential biomarker for tumor grading, metastasis, chemosensitivity, and prognosis of patients. In conclusion, FGFRL1 has a very unique molecular structure and important physiological functions. Further revealing its molecular function is expected to provide molecular targets for the diagnosis and treatment of human diseases.

## Abbreviations

ABS	Antley–Bixler syndrome
ALS	amyotrophic lateral sclerosis
Akt	protein kinase B
CDH	congenital diaphragmatic hernia
ENO1	enolase 1
ERK	extracellular signal-regulated kinase
ESCC	esophageal squamous cell carcinoma
FGF	fibroblast growth factor
FGFRL1	fibroblast growth factor receptor-like 1
FGFRs	fibroblast growth factor receptors
5′UTR	5′-non-coding sequence
HIF-1	hypoxia-inducible factor-1 (HIF-1)
HSPGs	heparan sulfate proteoglycans
IHC	immunohistochemical
LOH	loss of heterozygosity
MEK	mitogen-activated extracellular signal-regulated kinase
MSCs	mesenchymal stromal cells
NSCLC	non-small cell lung cancer
3′UTR	3′-non-coding sequence
OC	ovarian cancer
PC	pancreatic cancer
PCa	prostate cancer
PD	Parkinson’s disease
PI3K	phosphatidylinositol 3-kinase
SCLC	small-cell lung cancer
SHP-1	Src homology region 2 domain-containing phosphatase 1
SNP	single-nucleotide polymorphism
3′UTR	3′-non-coding sequence
WHS	Wolf–Hirschhorn syndrome
